# Characterization of LP-Z Lipoprotein Particles and Quantification in Subjects with Liver Disease Using a Newly Developed NMR-Based Assay

**DOI:** 10.3390/jcm9092915

**Published:** 2020-09-10

**Authors:** Shimpi Bedi, Erwin Garcia, Elias J. Jeyarajah, Irina Shalaurova, Maria Camila Perez-Matos, Z. Gordon Jiang, Robin P. F. Dullaart, Steven P. Matyus, William J. Kirk, James D. Otvos, W. Sean Davidson, Margery A. Connelly

**Affiliations:** 1 Center for Lipid and Arteriosclerosis Science, Department of Pathology and Laboratory Medicine, University of Cincinnati, Cincinnati, OH 45237-0507, USA; bedisi@ucmail.uc.edu (S.B.); davidswm@ucmail.uc.edu (W.S.D.); 2 Laboratory Corporation of America Holdings (LabCorp), Burlington, NC 27560, USA; garce14@labcorp.com (E.G.); eliasjey@gmail.com (E.J.J.); shalaui@labcorp.com (I.S.); matyuss@labcorp.com (S.P.M.); kirkw@labcorp.com (W.J.K.); otvosj@labcorp.com (J.D.O.); 3 Division of Gastroenterology & Hepatology, Beth Israel Deaconess Medical Center, Harvard Medical School, Boston, MA 02215, USA; mcperezmatos@thejianglab.org (M.C.P.-M.); zgjiang@bidmc.harvard.edu (Z.G.J.); 4 Department of Endocrinology, University of Groningen, University Medical Center Groningen, 9713 Groningen, The Netherlands; dull.fam@12move.nl

**Keywords:** lipoproteins, nuclear magnetic resonance spectroscopy, cholestasis, liver disease

## Abstract

Background: Lipoprotein particles with abnormal compositions, such as lipoprotein X (LP-X) and lipoprotein Z (LP-Z), have been described in cases of obstructive jaundice and cholestasis. The study objectives were to: (1) develop an NMR-based assay for quantification of plasma/serum LP-Z particles, (2) evaluate the assay performance, (3) isolate LP-Z particles and characterize them by lipidomic and proteomic analysis, and (4) quantify LP-Z in subjects with various liver diseases. Methods: Assay performance was assessed for linearity, sensitivity, and precision. Mass spectroscopy was used to characterize the protein and lipid content of isolated LP-Z particles. Results: The assay showed good linearity and precision (2.5–6.3%). Lipid analyses revealed that LP-Z particles exhibit lower cholesteryl esters and higher free cholesterol, bile acids, acylcarnitines, diacylglycerides, dihexosylceramides, lysophosphatidylcholines, phosphatidylcholines, triacylglycerides, and fatty acids than low-density lipoprotein (LDL) particles. A proteomic analysis revealed that LP-Z have one copy of apolipoprotein B per particle such as LDL, but less apolipoprotein (apo)A-I, apoC3, apoA-IV and apoC2 and more complement C3. LP-Z were not detected in healthy volunteers or subjects with primary biliary cholangitis, primary sclerosing cholangitis, autoimmune hepatitis, or type 2 diabetes. LP-Z were detected in some, but not all, subjects with hypertriglyceridemia, and were high in some subjects with alcoholic liver disease. Conclusions: LP-Z differ significantly in their lipid and protein content from LDL. Further studies are needed to fully understand the pathophysiological reason for the enhanced presence of LP-Z particles in patients with cholestasis and alcoholic liver disease.

## 1. Introduction

In the past two decades, nuclear magnetic resonance spectroscopy (NMR) has become a powerful tool to measure lipoprotein particle concentrations [1]. Accurate quantitation of lipoprotein classes and subclasses can be accomplished by NMR for three reasons: (1) each class of lipoproteins, very low-density lipoprotein (VLDL), low-density lipoprotein (LDL), and high-density lipoprotein (HDL), emits an NMR signal with a unique lineshape enabling their distinction from one another, (2) the amplitudes of these unique NMR signals are directly proportional to the number of particles emitting the signal thereby allowing for accurate quantification, and (3) there is a magnetic property specific to lipoproteins that causes the lipids in larger particles to emit signals that are characteristically different in shape and higher in frequency (chemical shift) from signals emitted by lipids in smaller particles [1,2,3]. The mathematical algorithms used to deconvolute the broad methyl signal within the NMR spectrum into the lipoprotein concentrations have improved and are now capable of capturing more lipoprotein subspecies than earlier algorithms [1,4,5,6].

Despite multiple optimizations to account for the diverse lipoproteins found in normolipidemic and dyslipidemic human samples, the deconvolution process continues to encounter specimens where lipoprotein concentrations cannot be accurately determined. In these situations, the mathematical signal generated by the algorithm fails to fit the acquired signal within defined cutoffs. It has been hypothesized that additional, unaccounted for signals may come from lipoproteins with different lipid compositions that are not captured by the existing algorithms. This offers an opportunity to identify novel lipoprotein species in human samples. Some of these lipoproteins have been described in the literature. In fact, patients with late-stage cholestatic liver diseases are known to have a distinct pattern of dyslipidemia in which circulating lipoproteins have abnormal compositions, electrophoretic mobility, and appearance [7]. One of these lipoproteins, called lipoprotein X (LP-X), has a multilamellar structure that is enriched in phospholipids and free cholesterol (FC) [8,9,10,11]. Its density is similar to that of LDL, but its size is in the VLDL range (40–100 nm) [8,9,10,11]. LP-X concentrations are increased in subjects with lecithin-cholesterol acyltransferase (LCAT) deficiency and primary biliary cholangitis (PBC) [8,9,10,11]. LP-X does not carry apolipoprotein (apo)B and has an aqueous core containing albumin. Unlike other lipoproteins, LP-X particles migrate toward the cathode during agarose gel electrophoresis [10,12,13,14]. Another lipoprotein species in patients with obstructive jaundice is lipoprotein Y (LP-Y) [7,14,15]. LP-Y is a large, triglyceride (TG)-enriched LDL particle that is 30–40 nm in diameter and carries apoB [7,14,15]. LP-Y particles are formed by deficiencies in LCAT and hepatic lipase (HL) activities and may be an intermediate-density lipoprotein (IDL) or a VLDL remnant [7,14,15]. Last, but not least, is a particle called “lipoprotein B” [14,15]. This particle was first described over 40 years ago as an LDL-sized particle with a diameter of 18–20 nm and a higher than normal TG and FC content [14,15]. Despite years of research, and likely due to the labor-intensive techniques conventionally required to characterize lipoproteins, few reports have been published regarding the characterization of these unique particles and a clear physiological and pathological picture of lipoprotein B has not emerged.

Through an in-depth evaluation of human specimens that failed to provide optimal lipoprotein quantitation using a standard algorithm, we rediscovered “lipoprotein B” and renamed it lipoprotein Z (LP-Z). Herein, we developed a novel NMR assay that can quantify LP-Z and LP-X, along with conventional lipoproteins. The aims of this study were to evaluate the ability of the assay to quantify LP-Z particles, isolate and characterize LP-Z by lipidomic and proteomic analysis, and qualitatively determine patient populations with elevated LP-Z.

## 2. Experimental Section

### 2.1. Development of an Assay for LP-Z Particle Quantification

**Figure 1:**
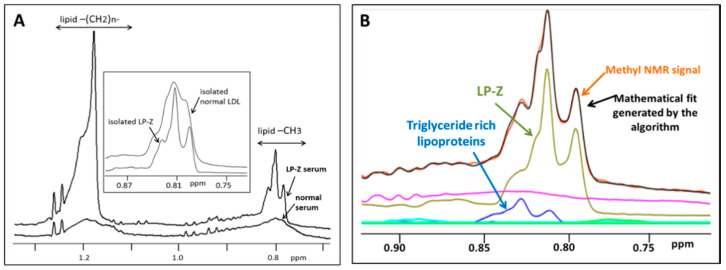
(**A**) Nuclear magnetic resonance (NMR) spectra from a normal serum sample and a serum sample from a subject with liver disease and a high bilirubin concentration “lipoprotein Z (LP-Z) serum”. The high bilirubin sample shows higher signals with differing lineshapes at both the methylene (lipid-CH_2_ group at approximately 1.2 ppm) and methyl (lipid-CH_3_ group at approximately 0.8 ppm) regions of the NMR spectrum. Inset: expanded view of the methyl group region showing the differences between the broader NMR signal from isolated low-density lipoproteins (LDL) versus the more distinct NMR signal from isolated LP-Z particles. These lineshapes are used as components in the deconvolution models that quantify the lipoprotein particle concentrations. (**B**) An NMR spectrum (orange line) demonstrating the signals from a subject with liver disease and a high bilirubin concentration in the methyl region. The black line shows the mathematically determined signal from the abnormal lipoprotein or LP-Z assay. The fact that these two lines superimpose means that the fit between the mathematically derived signal and the acquired signal is good. The signals from the triglyceride-rich particles are noted in blue; however, based on the lipoprotein analysis there were no signals observed from normal LDL particles in this sample. The signals from the LP-Z particles and serum proteins are represented by the green and purple lines, respectively.

The LP-Z particle assay was developed using NMR spectra from serum samples on a Vantera^®^ Clinical Analyzer [1,4,16]. NMR spectra collected for this assay, which includes quantification of LP-Z particles, as well as all of the other lipoprotein species, is acquired in the same fashion as spectra collected for the NMR LipoProfile^®^ test. Details of this procedure, including calibration of the Vantera Clinical Analyzer and controls used for lipoprotein quantification, have been described in detail in a previous publication [4]. Similar to quantification of other lipoprotein parameters, a software algorithm was developed that quantifies the methyl signals from all lipoprotein subclasses, centered around 0.8 ppm and spanning 463 data points of the NMR spectrum, using the spectral deconvolution method with non-negative least square analysis [1,4,16]. A library of spectral components was used, including those for the normal lipoproteins, collected from years of isolating lipoprotein subclasses from healthy subjects as well as from subjects with diseases that affect lipoproteins, and new components developed specifically for detecting LP-Z. The spectral components for the quantification of LP-Z particles were isolated from the NMR spectrum from serum of a cirrhotic patient waitlisted for a liver transplant [17]. A combination of ultracentrifugation and A-15 agarose gel chromatography was used to isolate lipoprotein particles in the d = 1.019–1.063 g/mL density range. NMR spectra from LP-Z particles (isolated using the isolation procedure described below) were obtained and multiple components (or lineshapes) were constructed for the deconvolution. Together, these components mimic the unique NMR signals from LP-Z particles in order to assist in their quantification, even when they are present in serum or plasma. The NMR signals from LP-Z span the NMR chemical shift range corresponding to small LDL (18.5–21.5 nm diameter). However, the signals for LP-Z and small LDL differ in their signal lineshapes, which allows the software algorithm to distinguish LP-Z from LDL and to accurately quantify both in the same sample (Figure 1A). The assay uses the LP4 deconvolution model that fits the components of the normal lipoproteins first, followed by a model that includes components for both LP-Z and LP-X. The deviation between the experimental and modelled lineshapes is calculated for both models and if substantial improvement in the fit is obtained, the LP-Z inclusive model is used to generate the lipoprotein concentrations, including results for LP-X and LP-Z when present. Figure 1B illustrates the composite NMR signal for LP-Z particles, in the background of the combined methyl signals from all lipoproteins, from a serum sample with a high bilirubin concentration. The signals from the TG-rich particles are shown in the graph. Based on the lipoprotein analysis, there were no signals present from LDL in this sample. Descriptions and diameter ranges of the normal lipoprotein, lipid, and apolipoprotein parameters reported from the LP4 algorithm have been published [5].

### 2.2. Evaluation of LP-Z Assay Performance

Five solutions (46 mg/mL; phosphate buffer, pH 7.4) of human serum albumin (Sigma-Aldrich, St. Louis, MO, USA) were prepared as blanks. The solutions were dialyzed through Slide-A-Lyzer 10 kDa cutoff cassettes (Thermo Scientific, Rockford, IL, USA) overnight at 4 °C to remove citrate. Five serum pools containing low LP-Z concentrations were tested to determine the limit of detection (LOD) and 8 serum pools were used to determine the limit of quantitation (LOQ) according to the Clinical and Laboratory Standards Institute (CLSI) guidelines [18]. The LOQ was determined at 20% coefficient of variation (CV). Linearity was evaluated by identifying and serially mixing serum pools that were predetermined by NMR to have low, medium, and high LP-Z levels [19]. Precision was determined using serum pools targeted at low and high ranges of LP-Z [20]. Within-run (intra-assay) precision was determined by analyzing each pool on one day (*n* = 20). The same pools were analyzed for 10 days, with two replicates twice-per-day (*n* = 40) to evaluate the within-laboratory (inter-assay) precision.

### 2.3. Isolation of LP-Z Particles from High Bilirubin Samples

De-identified, discarded serum specimens with high bilirubin (≥8 mg/dL) were collected from LabCorp’s central laboratory (Burlington, NC, USA). After applying the above-described assay, 8 specimens contained exclusively LP-Z, with no LP-X determined by agarose gel electrophoresis and NMR, or no LDL by NMR. These samples were combined into 4 pools. All specimens had very low levels of HDL-C (<10 mg/dL) and high bilirubin concentrations (12.2–27.2 mg/dL). Separately, serum specimens with normal bilirubin levels without any LP-Z particles, as determined by NMR, were chosen as control specimens. The LP-Z assay was run on all six pooled samples (4 with high and 2 with normal bilirubin concentrations). From the remainder, 3 mL was used for lipoprotein isolations. Specimens were placed in 4.7 mL centrifuge tubes (Beckman Coulter, Pasadena, CA, USA) and solid sodium bromide (NaBr) (EMD Millipore, Bellerica, MA, USA) was added to adjust the density to 1.019 g/mL. The contents were then layered with 1 mL of d 1.019 g/mL phosphate buffer (pH7.4) and spun in a Beckman Coulter Optima TLX Ultracentrifuge in a TL-110 fixed angle rotor (Beckman Coulter, Pasadena, CA, USA) at 45,000 rpm for 20 h at 4 °C. The centrifuge tubes were sliced and the top 1 mL fraction was discarded. The bottom fraction was then adjusted to d 1.063 g/mL, layered with 1 mL buffer of the same density, and centrifuged at 50,000 rpm for 24 h at 4 °C. The tubes were sliced and the top 1 mL fractions containing lipoproteins in the 1.019–1.063 g/mL density range were harvested. This fraction contains the LP-Z and/or LDL particles. The isolated fractions were placed in Centricon tubes with a molecular weight cut off (MWCO) of 10,000 (MilliporeSigma, Burlington, MA, USA) for buffer exchange against d1.006 g/mL KBr at pH7.4 through ultrafiltration. The fractions were concentrated 3-fold to a final volume of 1 mL each. An aliquot was used to collect another set of NMR spectra as described above. In another isolation and purification, LP-Z was obtained from serum specimens (*n* = 4) with high bilirubin and LDL from normal serum specimens (*n* = 4). Aliquots were used to perform the lipidomic and proteomic analysis.

### 2.4. Agarose Gel Electrophoresis and Staining

Human plasma was analyzed by agarose gel electrophoresis. Briefly, 10 µL of plasma sample was loaded in the agarose gel. The gel was run on Barbital buffer for 1 h at 100 mV. Two gels per sample were run; one stained with Sudan Black for neutral lipids, and the other with Filipin for FC. For Sudan Black staining, the gel was fixed and put in a preheated oven at 70 °C overnight. The next day, the gel was stained for 15 min with Sudan Black solution. For Filipin staining, the gel was washed with 1X phosphate buffered saline (PBS), 0.1% sodium azide, and stained overnight with a 1% fresh Filipin solution at 4 °C. The next day, the gel was washed 3 times with 1X PBS 0.01% sodium azide for 1 h at room temperature, and imaged with a ChemiDoc Imaging System (Biorad, Hercules, CA, USA).

### 2.5. Lipid and ApoB Content of Isolated Lipoproteins

The TG, total cholesterol (TC), FC, phospholipid, and cholesteryl ester (CE) content of the lipoproteins was determined using kits from Wako Diagnostics (Mountain View, CA, USA) as per manufacturer’s instructions. A Tina-quant assay was used to quantify apoB levels on a Cobas c501 (Roche Diagnostics, Basel, Switzerland).

### 2.6. Targeted Lipidomic and Metabolomic Analysis

A MxP^®^ Quant 500 kit (Biocrates Life Sciences, Innsbruck, Austria) was used for the quantification of lipid of various biochemical classes. Lipids and hexoses were measured by flow injection analysis-tandem mass spectroscopy (FIA-MS/MS) using Xevo^®^ TQ-S instrument (Waters, Milford, MA, USA) with an electrospray ionization source, and small molecules were measured by liquid chromatography-tandem mass spectroscopy (LC-MS/MS) using the same Xevo TQ-S instrument. Briefly, a 96-well based sample preparation device was used to quantitatively analyze the lipid profile in the samples. This device consists of inserts that have been impregnated with internal standards, and a predefined sample amount was added to the inserts. Next, a phenyl isothiocyanate solution was added to derivatize some of the analytes (amino acids) and after the derivatization was completed, the target analytes were extracted with an organic solvent, followed by a dilution step. The obtained extracts were then analyzed by FIA-MS/MS and LC-MS/MS methods using multiple reaction monitoring (MRM) to detect the analytes. Data were quantified using appropriate mass spectrometry software (Analyst^®^, Sciex, Framingham, MA, USA).

### 2.7. Protein Delipidation and Solubilization

Isolated LP-Z or LDL samples were delipidated by organic solvent as previously described [21] with modifications. Briefly, samples were dialyzed into 50 mmol/L ammonium bicarbonate (NH_4_HCO_3_) at pH 8.1 and lyophilized to dryness. Lipids were extracted by addition of 1.0 mL of ice-cold chloroform, MeOH (2:1 *v/v*), and incubation on ice for 30 min. Ice-cold methanol was added to a final chloroform, MeOH ratio of 1:1 (*v/v*), and protein was pelleted by centrifugation at 8000× *g* for 30 min at 4 °C. The solvent was carefully decanted, and the protein was resuspended in 2.0 mL of ice-cold methanol and mixed by vortexing and sonication. Protein was pelleted by centrifugation and resuspended by intense vortexing and repeated aspiration using 100 μL of cold 20% acetic acid/80% 6 M guanidine hydrogen chloride (HCl), 0.2 M ammonium bicarbonate. Samples were sequentially dialyzed against 6 M guanidine HCl/6 M urea/0.2 M ammonium bicarbonate, 5 M urea/0.2 M ammonium bicarbonate and finally in 2 M urea/0.2 M ammonium bicarbonate containing buffers [22]. Protein content in the soluble fraction was measured by Bradford protein assay.

### 2.8. Tryptic Protein Digestion

Sequencing grade trypsin (Promega, Madison, WI, USA) was added (2.5% of protein by weight) to 50 µg of delipidated protein sample. Digestion proceeded for 16 h at 37 °C. Additional trypsin (2.5% of protein by weight) was added and samples were digested for an additional 2 h at 37 °C. Urea was removed from the peptides using C18 spin columns (Thermo Fisher Scientific, Waltham, MA, USA) and samples were lyophilized to dryness. Dried samples were stored at −20 °C until mass spectrometry (MS) analysis.

### 2.9. Proteomic Analysis

Proteomic analysis was performed by a quadrupole time-of-flight (Q-TOF) LC/MS system using 10 µg of digested peptides. Peptides were resuspended in 0.1% formic acid/H_2_O 0.1/99.9 (*v/v*) and 10 µL of each sample was loaded onto a 50 cm C18 column attached to the mass spectrometer. The separation was carried out using an Agilent 1290 Infinity UHPLC coupled to a 6550 iFunnel quadrupole time-of-flight LC/MS equipped with a dual-spray Agilent Jet Stream electrospray ionization source (Agilent Technologies, Santa Clara, CA, USA). Reference mass ions were delivered using an Agilent 1260 Infinity Isocratic Pump (G1310B) using a 1 in 100 flow splitter (p/n G1607-60000). The Q-TOF LC/MS instrument was operated with Agilent MassHunter Data Acquisition software, rev. B.05.01, (Agilent Technologies, Santa Clara, CA, USA) in 2 GHz extended dynamic range mode with positive or negative ionization with two different methods. In target MS/MS acquisition, a data rate of three scans/s in MS/MS mode was used. Peptides were eluted by an acetonitrile gradient.

Peptide spectral data were searched against the UniProtKB/Swiss-Prot Protein knowledgebase (release February 2016, 550,552 sequences) for Homo sapiens (20,273 sequences) using Mascot (2.2.07). Data were constrained to tryptic digestion with a maximum of three missed cleavages. Carbamidomethylation was set as a fixed modification and methionine oxidation as a variable modification. Peptide and MS/MS mass tolerance was ±0.15 Da. Scaffold (v 4.3.4) was used for MS/MS-based peptide validation using X! Tandem (2010.12.01.1). Proteins and peptides were constrained to >99.9% and 95% identification probability, respectively. Additionally, proteins were only accepted if they contained a minimum of two unique peptides [23]. The proteomics data were normalized to account for differences in the ionization efficiency of peptides between samples. Spectral counts of identified proteins were summed and the total counts were normalized across samples to recalculate the normalized spectral counts for each protein. A p-value was obtained for each protein using a paired *t*-test in SigmaPlot to determine the statistical significance of the proteomic differences. Ion intensity-based quantitative changes in protein abundance between samples were generated using Skyline. Peptide peak areas under the curve were individually normalized to each of the five most efficiently ionized (most intense peak areas) apoB peptides to estimate relative differences in protein abundance.

To ensure the optimal performance of the system, quality control was incorporated by running digested apoA-I peptide samples at the beginning and conclusion of the sample run. Blank runs (buffer) were incorporated between two consecutive sample runs to eliminate carry-over of contaminants.

### 2.10. Polyacrylamide Gel Electrophoresis

Serum samples as well as samples of purified LP-Z or LDL were reduced with 3 mM β-mercaptoethanol and analyzed by sodium dodecyl sulfate-polyacrylamide gradient gel electrophoresis (SDS-PAGE) (4–15%) (BioRad, Hercules, CA, USA) until bromophenol blue dye front reached the bottom of the gel. The gel was stained for protein with Coomassie brilliant blue (GE Healthcare, Chicago, IL, USA).

### 2.11. Quantification of LP-Z in Samples from Subjects with Liver Disease or Controls

Ethylenediaminetetraacetic acid (EDTA) plasma samples from subjects with various liver diseases such as PBC (*n* = 11), primary sclerosis cholangitis (PSC) (*n* = 11), autoimmune hepatitis (AIH) (*n* = 19), alcoholic liver disease (ALD) (*n* = 47) were obtained from Discovery Life Sciences (Huntsville, AL, USA). For the controls, apparently healthy adult men and women (ages 18 to 84 years) were recruited as described [4]. Fasting and non-fasting serum specimens were included in this study (*n* = 769). For the metabolic syndrome and/or type 2 diabetes mellitus (T2DM) cohort, subjects were aged > 18 years as previously reported (*n* = 138) [24]. All studies were conducted in accordance with the Helsinki declaration. Samples from all studies were collected under a protocol that was cleared by an Institutional Review Board and all donors signed consent forms. For the hypertriglyceridemia samples (TG ≥ 500 mg/dL), digitally stored NMR spectra from 278,643 serum samples were reanalyzed using the LP4 algorithm. Of these, 1970 samples with hypertriglyceridemia (≥500 mg/dL TG) were included in this analysis.

### 2.12. Statistical Analysis

Statistical analysis was performed using JMP version 13.1.0 (SAS Institute, Cary, NC, USA). The *t*-test for unpaired samples was used to determine statistical significance of the lipidomic data. Two-sided *p*-values < 0.05 were considered significant. The level of significance was corrected according to Bonferroni to account for multiple comparisons.

## 3. Results

### 3.1. Analytical Performance of the LP-Z NMR Assay

**Figure 2:**
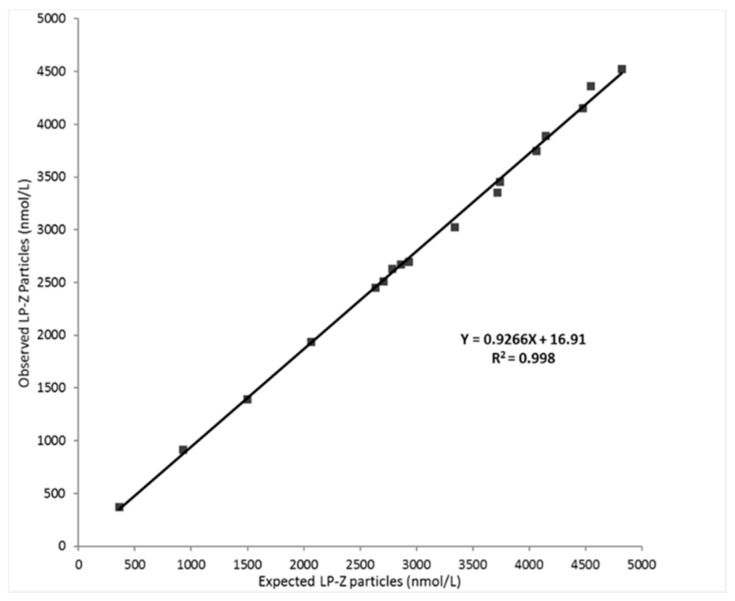
Linearity of expected versus observed LP-Z particles in serum. Linearity was evaluated by identifying and serially mixing serum pools that were predetermined by NMR to have low, medium, and high LP-Z levels.

The limit of blank (LOB), the LOD and the LOQ were determined to be 0, 295, and 352 nmol/L, respectively. Linearity was demonstrated between 350 and 4485 nmol/L with a correlation coefficient (R^2^) of 0.998 (Figure 2).

**Figure 3:**
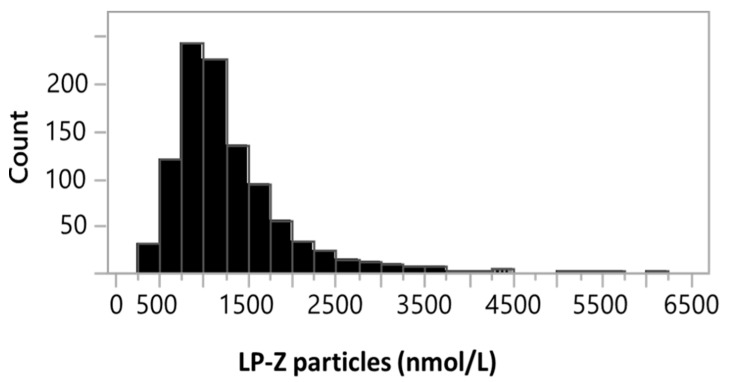
Distribution of LP-Z particles in 990 subjects with quantifiable LP-Z particles out of a total of 278,643 serum samples tested in the clinical NMR laboratory.

Based on the linearity and LOQ data, the reportable range for LP-Z is 352–4485 nmol/L. The intra-assay and inter-assay precisions were 2.5–6.3% and 3.8–4.2%, respectively. As LP-Z was not detected in apparently healthy adults, reference intervals were not determined. However, the frequency of samples containing LP-Z particles in the general population was determined to be 0.36% by analyzing 278,643 digitally stored NMR spectra acquired from samples sent for the NMR LipoProfile^®^ test. The distribution of LP-Z concentrations in the 990 subjects with quantifiable LP-Z particles can be found in Figure 3. Taken together, these data provide evidence that the performance of the LP-Z assay was sufficient for clinical laboratory testing.

### 3.2. Characterization of Isolated LP-Z Particles

**Figure 4:**
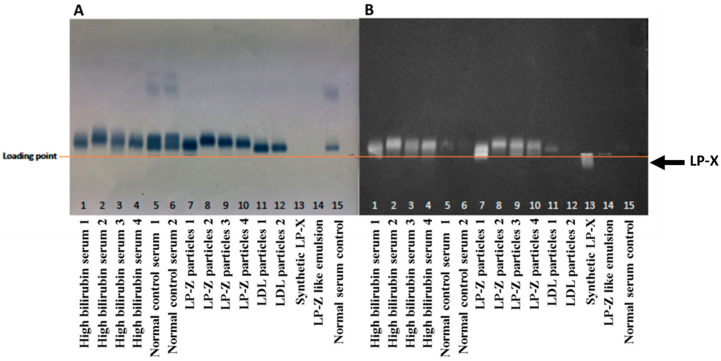
Agarose gel electrophoresis of serum and isolated lipoprotein particle samples to reveal migration of lipoproteins, such as lipoprotein X (LP-X), toward the cathode. (**A**) Sudan Black lipid stained agarose gel and (**B**) Filipin free cholesterol stained agarose gel.

Given that LP-Z were originally identified in patients with obstructive jaundice, we collected serum samples with bilirubin concentrations ≥ 8 mg/dL. For comparison, serum samples from presumably healthy individuals (i.e., normal bilirubin levels and normal lipoprotein profiles determined by NMR) were collected. An NMR lipoprotein analysis was performed for each sample before particle isolation. From these, specimens with high concentrations of LP-Z particles, but few or no small, medium, or large LDL particles by NMR were chosen for further characterization (Table 1). These samples had low HDL concentrations compared to the normal serum samples (Table 1) and no LP-X as assessed by agarose gel electrophoresis (Figure 4) as well as by NMR.

For the characterization of LP-Z particles by lipid and proteomic analyses, lipoproteins in the LDL density range (1.019–1.063 g/mL) were isolated by ultracentrifugation. The isolated particles were subjected to lipid and protein analysis to quantify the amount of protein, phospholipids, TG, FC, and CE in LP-Z compared to LDL particles. When normalized by mass (Table 2), results revealed that LP-Z contained similar percentages of protein per particle compared to LDL. However, LP-Z contained 28% more FC, 2.6-fold more TG, and 4.2-fold less CE than LDL particles. When normalized by the number of moles, similar results were observed. Therefore, the ratios of FC/CE and TG/CE were increased in LP-Z compared to LDL. Results were similar when 4 additional samples of isolated LDL were added to the analysis.

**Figure 5:**
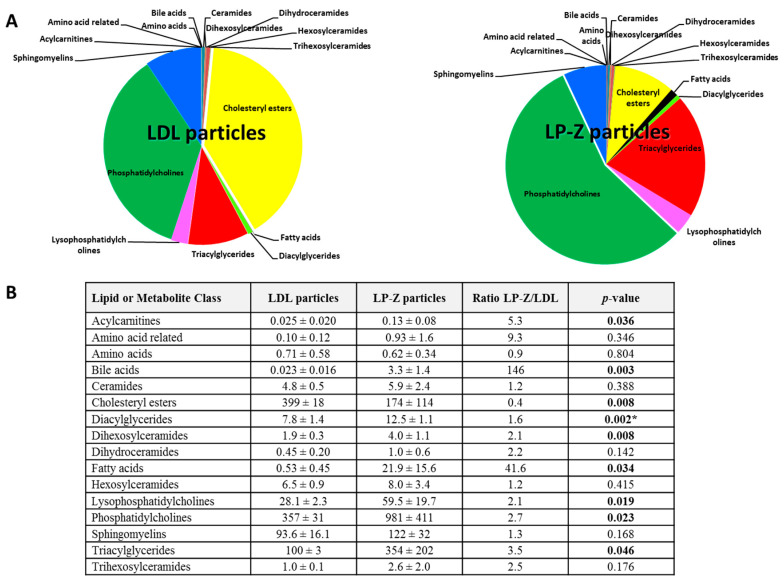
Targeted lipidomic and metabolomic data for isolated LP-Z (*n* = 4) versus LDL particles (*n* = 4). (**A**) Data expressed as mean (µM) ± standard deviation. Values were normalized to apolipoprotein B content. *p* values < 0.05 are in bold. (**B**) The *p*-value threshold with Bonferroni correction = 0.003. * Significant when taking into account Bonferroni correction. Abbreviations: LDL, low-density lipoprotein.

A targeted lipidomics/metabolomics analysis was performed in order to further define the differences in lipid subspecies between the particles. Some lipids (e.g., CE 14:0, CE 14:1, dodecanoic acid, myristic acid, palmitic acid, stearic acid) were absent in either LP-Z or LDL particles. When normalized to apoB concentrations, bile acids were significantly higher in LP-Z than LDL (*p* = 0.003), while amino acid concentrations were the same in both (*p* = 0.804). Additionally, CE were lower (*p* = 0.008), while acylcarnitines, free fatty acids (FFA), diacylglycerides, TG, dihexosylceramides, lysophosphatidylcholines, and phosphatidylcholines were higher in LP-Z than in LDL (*p* = 0.008–0.05, Figure 5). A complete list of lipids and the differences between LP-Z and LDL particles can be found in Supplementary Table S1.

**Figure 6:**
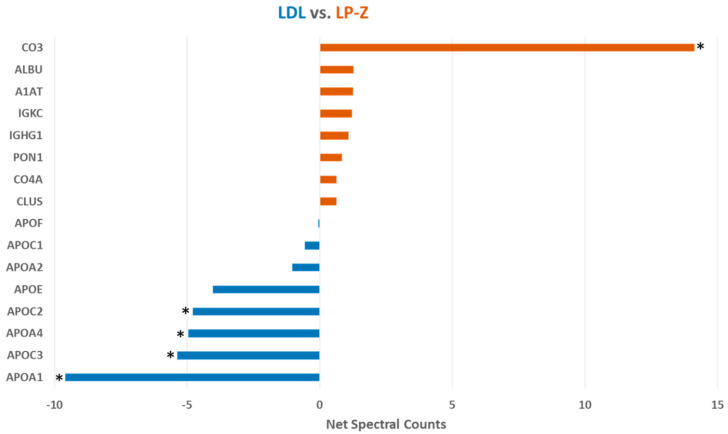
Proteomic composition of isolated LP-Z and LDL particles. * *P* value < 0.05 determined by *t*-test using Skyline Mass Spectrometry 1 (MS1) data normalized to apoB peptides. LDL particle data are in blue (*n* = 6) and LP-Z particle data are in red (*n* = 4). Abbreviations: LDL, low-density lipoproteins; CO3, complement C3; ALBU, albumin; A1AT, alpha1-antitrypsin; IGKC, immunoglobulin kappa constant antibody light chain; IGHG1, immunoglobulin heavy constant antibody gamma 1 chain; PON1, paraoxonase 1; CO4A, complement C4-A; CLUS, clusterin; APOF, apolipoprotein F; APOC1, apolipoprotein C1; APOA2, apolipoprotein A-II; APOE, apolipoprotein E; APOC2, apolipoprotein C2; APOA4, apolipoprotein A-IV; APOC3, apolipoprotein C3; APOA1, apolipoprotein A-I.

**Figure 7:**
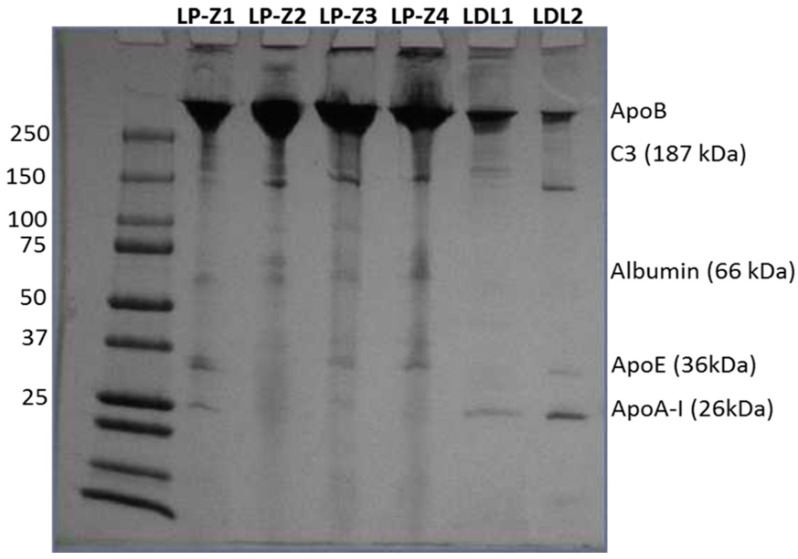
Polyacrylamide gel electrophoresis of isolated LP-Z (LP-Z1–Z4) and LDL (LDL1, LDL2) particles.

The amount of apoB per isolated LP-Z particle was calculated using chemistry-based assays for apoB concentrations and NMR to determine the number of particles. The number of apoB molecules per particle was determined to be one. Qualitative proteomic analysis using net spectral counts determined by mass spectrometry and normalized to multiple apoB peptides revealed that LP-Z carry less apoA-I, apoC3, apoA-IV and apoC2 and more complement C3 than LDL (Figure 6). These conclusions were confirmed using more sophisticated Mass Spectrometry 1 (MS1) full scan filtering approaches (Skyline) normalized to multiple apoB peptides (not shown). In addition, the quantitative analysis confirmed that LP-Z tended to have more immunoglobulin kappa constant antibody light-chain (IGKC), paraoxonase-1 (PON-1) and clusterin/apoJ than LDL particles. Polyacrylamide gel electrophoresis confirmed some of the differences in proteins observed by the mass spec analysis (Figure 7).

### 3.3. Quantification of LPZ Particles in Serum of Subjects with Various Liver Diseases

Given that high levels of LP-Z particles are found in subjects with high bilirubin concentrations, a condition common in liver diseases, LP-Z levels were measured in subjects with various liver diseases. Plasma samples were tested from subjects with PBC, PSC, AIH, ALD, metabolic syndrome or T2DM, and hypertriglyceridemia, and LP-Z concentrations were compared to those from healthy adult volunteers (Table 3). Most of the serum samples, including the healthy adults, as well as patients with PBC, PSC, and AIH had no measurable levels of LP-Z particles (Table 3). However, 9 out of 47 of the subjects with ALD and 108 out of 1970 with hypertriglyceridemia exhibited appreciable levels of LP-Z.

## 4. Discussion

This study is the first to report a newly developed NMR assay for the quantification, as well as to comprehensively characterize the lipidomic and proteomic content, of LP-Z particles. The initial lipid analysis revealed that LP-Z have 2.6-fold higher TG and 4.2-fold lower CE than normal LDL particles. They also contained more FC. Therefore, the ratios of FC/CE and TG/CE were considerably increased in LP-Z compared to LDL. Moreover, a targeted lipidomic analysis revealed that CE were lower while acylcarnitines, FFA, diacylglycerides, TG, dihexosylceramides, lysophosphatidyl-cholines and phosphatidylcholines were higher in LP-Z than in LDL. With Bonferroni correction for multiple comparisons, only diacylglycerides remained statistically significantly higher. This may be due to the small number of samples coupled with the lipid heterogeneity within the particles. However, the data remained consistent with the initial lipid analysis despite being from different sets of particles from different donor samples.

Previous studies have shown differing results for lipid analyses in patients with ALD [25]. In the Sabesin et al. study, subjects with ALD had decreased CE and increased TG but exhibited a 2-fold higher FC content than the isolated particles in the current study [25]. While the FC content of the LP-Z and LDL did not appear to be very different when measured chemically (Table 2), Filipin staining of the particles in the agarose gel suggested that the FC content of LP-Z was much higher than that of LDL (Figure 4). Additionally, the differences in the FC content of the particles between the studies could be due to the stage of liver disease at the time the samples were obtained. There was significant heterogeneity in the FC, TG and CE content in the isolated particle preparations as well as varied bilirubin and LP-Z concentrations present in the serum samples that may be related to the stage of liver disease and/or comorbidities that may have been present in the patients whose samples were collected for this study. However, a complete clinical characterization of the donors was not possible using current samples, and more studies are needed to further define pathological conditions associated with LP-Z accumulation.

While there was heterogeneity in the concentrations of the lipid species, it is clear that LP-Z had higher concentrations of FC, TG, diacylglycerides, phosphatidylcholines, and lower CE than LDL. Taken together, these data are consistent with previous descriptions of LP-Z particles in the literature [14,15,26]. These lipid changes may be explained by differences in the levels and activities of key proteins that regulate lipoprotein metabolism in subjects with liver disease. For example, patients with severe liver disease have lower circulating LCAT, cholesteryl ester transfer protein (CETP) and higher HL than healthy subjects [15,25,27], raising the possibility that defective lipoprotein remodeling and catabolism may account for the appearance of LP-Z. This is in contrast to elevated LCAT activity in subjects with non-alcoholic fatty liver disease (NAFLD), normal bilirubin levels and mildly elevated liver enzymes [28]. While enzyme activities were not measured in this study, it is likely that reductions in LCAT activity contribute to the development of LP-Z. Previous studies support the hypothesis that LCAT, CETP, HL, apoA-I and apoA-IV are reduced in patients with liver disease and cirrhosis [7,15,26,27,29]. Typical of patients with liver diseases, the high bilirubin samples exhibited low HDL-C and HDL particles. However, they did not have any LP-X suggesting that LP-Z were not formed as a consequence of a complete loss of LCAT activity.

**Figure 8:**
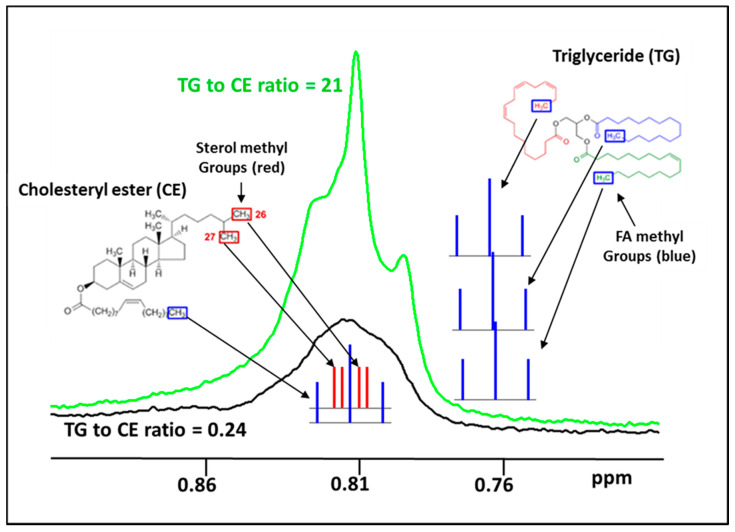
Illustration of the two types of methyl groups that contribute to the detected LDL signal: (1) “fatty acid (FA) methyls” (blue) at the termini of the three triglyceride (TG) fatty acyl chains and the cholesteryl ester (CE) acyl chain, and (2) the C26 and C27 “sterol methyls” (red) of the CE. In normal LDL (black line), both types of methyl groups make substantial contributions to the overall NMR signal lineshape. As the ratio of TG/CE increases in LP-Z particles, the lineshape gets sharper and taller as it is dominated by the increase in the signals from the FA methyl triplets (green).

The NMR signals, which are used for the quantification of lipoprotein particles, arise from the methyl group protons on the lipid molecules within the lipoproteins and the lineshapes of these signals are dependent on the environment of the protons within the particle [1]. Two types of methyl groups contribute to the detected LDL signal: (1) “fatty acid (FA) methyls” at the termini of the TG fatty acyl chains and CE fatty acyl chains, and (2) the C26 and C27 “sterol methyls” of CE (Figure 8). The chemical shifts of these two types of methyl groups are identical, so their signals superimpose to create the observed LDL (or LP-Z) lineshape. Due to spin coupling with the adjacent proton(s), the multiplet structures of their signals are very different—a triplet and doublets, respectively (Figure 8). In LDL, both types of methyl groups make contributions to the overall LDL lineshape, which tends to be broad with indistinct features because the multiplets fill in the spaces between the triplets, and also the result of overlapping signals from such methyls in different microenvironments within the particle. As the ratio of TG/CE increases, the particle lineshape becomes dominated by FA methyl triplets and gets sharper in appearance. The increased FA methyl groups from TG, diacylglycerides and FFA likely contribute to the sharper LP-Z lineshape. The observed LP-Z lineshapes are therefore an extension of the trend toward a FA methyl triplet-dominated signal (Figure 8). This differing lineshape is the basis for the ability of the assay to quantify LP-Z in serum or plasma specimens, even when large and small LDL are present.

The lipidomic analysis revealed that LP-Z have an overall higher concentration of lipids than LDL particles. The size and density of LP-Z, however, remain similar to LDL. Therefore, it is likely that the lipids within LP-Z particles are packed more tightly than in LDL. FC levels were higher in LP-Z as were FFA levels. Cholesterol and some of the more saturated FA are known to pack more tightly in membranes than other lipids, which may explain why LP-Z are not larger in size despite the increased lipid content compared to LDL.

Proteomic analysis revealed that LP-Z have one apoB per particle, however they carry less apoA-I apoC3, apoA-IV, and apoC2 and more complement C3 than LDL. LP-Z also tended to have more IGKC, PON-1 and clusterin/apoJ than LDL particles. Proteins that are usually associated with VLDL and HDL have been reported to be associated with LDL particles by proteomic analysis [30,31,32]. However, the current results suggest that LP-Z are present in subjects with higher circulating inflammatory proteins and/or are more pro-inflammatory in nature. Moreover, higher complement C3 levels have been reported in subjects with chronic liver disease [33]. Some of these proteomic changes may be explained by the increased circulating bile acids observed in the subjects with high levels of LP-Z. High levels of bile acids may lead to the activation of the farnesoid-X receptor (FXR), an intracellular bile acid-sensing transcription factor that regulates hepatic synthesis and transport of bile acids [34,35]. FXR regulates cholesterol, lipid and carbohydrate metabolism and inflammation, and FXR activation leads to reductions in apoA-I and apoC3 and an increase in complement C3 [34,35,36,37,38].

LP-Z particles were originally described in patients with obstructive jaundice [14]. Obstructive jaundice has become a rare diagnosis due to advances in hepatobiliary surgical techniques and advanced endoscopy for the biliary tree. Interestingly, among the limited samples that were tested, LP-Z was not found in PBC or PSC, conditions known to cause cholestasis. Our limited survey does not rule out the possibility that LP-Z is present in patients with late-stage intrahepatic cholestatic liver disease and future studies will include such samples. However, we did detect high levels of LP-Z in 19.1% of patients with ALD and 5.5% of those with hypertriglyceridemia. LP-Z was not detected in patients with metabolic syndrome or T2DM, conditions that often coexist with hyper-triglyceridemia. Of note, alcoholism is another common cause for hypertriglyceridemia [39]. Hence, our preliminary study suggests that LP-Z are likely associated with ALD. Among the spectrum of ALD, alcoholic hepatitis and alcoholic cirrhosis can present with hyperbilirubinemia. Interestingly, a study by Sabesin and colleagues reported four patients with acute ALD presenting with hepatomegaly, hepatic steatosis, intrahepatic cholestasis and altered circulating lipoproteins [25]. The clinical description of these patients fits with the diagnosis of alcoholic hepatitis. Further analyses showed that all four patients had lipoproteins deficient in CE, but elevated FC and TG, in keeping with the characteristics of LP-Z. In both cases of obstructive jaundice and alcoholic hepatitis, it has been suggested that functional deficiency of LCAT could be responsible for the altered lipid composition in LP-Z [15,25]. This hypothesis could explain the altered FC/CE ratio in LP-Z, but should not account for the higher levels of TG. What remains unclear is how LCAT becomes deficient in both obstructive jaundice and alcoholic hepatitis. Unfortunately, the early insights in the 1970s were largely forgotten by the scientific community and not many mechanistic studies have been pursued since then. Since ALD is increasingly prevalent and has become the major driver for increased mortality in the United States, the biology relevant to LP-Z, which could be unique to alcoholic hepatitis, warrants further investigation [40].

## 5. Conclusions

In conclusion, LP-Z particles differ significantly in their lipid and protein content from LDL particles. An NMR based assay to quantify LP-Z particles was developed, which exhibits performance characteristics consistent with use for the interrogation of clinical samples. Further studies are needed to fully understand the pathophysiological reason for the enhanced presence of LP-Z particles in patients with cholestasis and/or alcoholic liver disease.

## Figures and Tables

**Table 1:** Concentrations of NMR-measured lipid and lipoprotein parameters in samples from normal subjects (*n* = 2) versus subjects with high bilirubin (*n* = 4).

Lipid Related Parameters	Normal Control 1	Normal Control 2	High Bilirubin 1	High Bilirubin 2	High Bilirubin 3	High Bilirubin 4
Bilirubin (mg/dL)	ND	ND	22.2	13.4	18.9	24.6
TG (mg/dL)	48.0	59.9	145.9	347.8	443.8	771.2
TC (mg/dL)	121.3	189.3	90.7	194.8	265.6	343.5
LDL-C (mg/dL)	77.1	82.9	80.1	169.3	98.7	84.3
HDL-C (mg/dL)	38.4	102.3	6.4	4.9	7.7	6.7
Apo A-I (mg/dL)	102.0	169.4	19.7	14.7	23.1	21.6
Apo B (mg/dL)	60.8	57.1	175.0	364.0	368.3	304.1
TRL-P (nmol/L)	26.8	18.6	16.3	81.5	985.8	651.8
VL-TRL-P (nmol/L)	0.1	0.0	0.0	0.0	0.0	3.5
L-TRL-P (nmol/L)	0.0	0.0	0.0	0.0	2.2	6.6
M-TRL-P (nmol/L)	4.5	1.5	16.3	81.5	63.0	369.2
S-TRL-P (nmol/L)	22.2	17.1	0.0	0.0	0.0	0.0
VS-TRL-P (nmol/L)	0.0	0.0	0.0	0.0	920.6	272.5
LDL-P (nmol/L)	1112	1170	3427	7026	5693	4531
L-LDL-P (nmol/L)	220.9	0.0	0.0	0.0	0.0	123.8
M-LDL-P (nmol/L)	695.3	429.3	0.0	0.0	0.0	0.0
S-LDL-P (nmol/L)	195.6	740.2	458.8	1085.2	0.0	0.0
LP-Z (nmol/L)	0.0	0.0	2968	5941	5693	4407
HDL-P (µmol/L)	19.4	20.8	3.8	4.9	7.7	6.3
L-HDL-P (µmol/L)	0.9	7.2	0.3	0.0	0.0	0.0
M-HDL-P (µmol/L)	4.1	6.2	0.0	0.0	0.0	0.0
S-HDL-P (µmol/L)	14.4	7.4	3.6	4.9	7.7	6.3
TRLZ (nm)	36.4	37.7	42.0	40.3	50.1	51.0
LDLZ (nm)	21.1	20.1	19.0	19.0	19.9	22.4
HDLZ (nm)	8.8	10.6	8.3	7.4	7.4	7.5

Abbreviations: Apo A-I, apolipoprotein A-I; Apo B, apolipoprotein B; HDL-C, high-density lipoprotein cholesterol; HDL-P, high-density lipoprotein particle number; HDLZ, high-density lipoprotein particle size; L-, large; LDL-C, low-density lipoprotein cholesterol; LDL-P, low-density lipoprotein particle number; LDLZ, low-density lipoprotein particle size; M-, medium; S-, small; TC, total cholesterol; TG, triglycerides; TRL-P, total triglyceride rich lipoprotein particle number; TRLZ, triglyceride rich lipoprotein particle size; VL-, very large; VS-, very small.

**Table 2:** Percentages of lipid and protein by mass in the LDL fraction isolated from subjects with normal (*n* = 2) or high bilirubin concentrations (*n* = 4).

Mass % Composition	Normal Control 1	Normal Control 2	High Bilirubin 1	High Bilirubin 2	High Bilirubin 3	High Bilirubin 4
Protein	21.9	20.5	21.4	22.6	23.9	22.2
Phospholipids	20.5	21.3	26.6	23.3	23.9	26.7
Triglycerides (TG)	11.4	15.8	29.2	44.1	32.7	34.6
Free cholesterol (FC)	7.4	5.4	8.6	6.2	8.6	9.2
Cholesteryl esters (CE)	38.8	37.0	14.2	3.8	10.8	7.4
FC/CE ratio	0.2	0.1	0.6	1.6	0.8	1.2
TG/CE ratio	0.3	0.4	2.1	11.6	3.0	4.7

Abbreviations: CE, cholesteryl ester; FC, free cholesterol; LDL, low-density lipoprotein; TG, triglycerides.

**Table 3:** Plasma/serum levels of LP-Z particles, along with triglyceride (TG) and total cholesterol (TC) levels in normal healthy adults versus subjects with various liver and metabolic diseases.

Subjects (# of Samples Tested)	TG (mg/dL)	TC (mg/dL)	LP-Z (nmol/L)
Normal healthy adults (769)	148 ± 94	186 ± 36	0.0
Primary biliary cholangitis (PBC) (11)	113 ± 30	173 ± 21	0.0
Primary sclerosing cholangitis (PSC) (11)	131 ± 61	179 ± 48	0.0
Autoimmune hepatitis (AIH) (19)	116 ± 64	170 ± 48	0.0
Alcoholic liver disease (ALD) (47)	99 ± 72	133 ± 46	1550 ± 1257 *
T2DM and metabolic syndrome (138)	172 ± 124	192 ± 31	0.0
Hypertriglyceridemia (≥500 mg/dL TG) (1970)	730 ± 266	273 ± 58	1690 ± 772 **

Data expressed as mean ± standard deviation. * 9 out of 47, ** 108 out of 1970 samples. Abbreviations: T2DM, type 2 diabetes mellitus; TG, triglycerides; TC, total cholesterol.

## Supplementary Materials

The following are available online at https://www.mdpi.com/2077-0383/9/9/2915/s1, Table S1: Concentrations of lipids and metabolites for isolated LDL (*n* = 4) versus LP-Z particles (*n* = 4).
