# Supplementary material for: Characterization of LP-Z Lipoprotein Particles and Quantification in Subjects with Liver Disease Using a Newly Developed NMR-Based Assay
Source: J Clin Med. 2020 Sep 10;9(9):2915. doi: 10.3390/jcm9092915 (PMC7564541; doi:10.3390/jcm9092915)
Supplement: Supplementary file 1 [file jcm-09-02915-s001.pdf]

Supplementary material

Supplementary Table 1. Concentrations of lipids and metabolites for isolated LDL (n=4) versus LP-Z particles (n=4).

Lipids/Metabolites	LDL	LP-Z	Ratio LPZ/LDL	p-value
Malonylcarnitine	0.000 ± 0.000	0.005 ± 0.010	*5.0*	0.356
Hydroxypropionylcarnitine	0.000 ± 0.000	0.003 ± 0.006		0.356
Propenylcarnitine	0.000 ± 0.000	0.006 ± 0.007	*6.0*	0.170
Butyrylcarnitine	0.000 ± 0.000	0.006 ± 0.012	*6.0*	0.356
Pimelylcarnitine	0.009 ± 0.018	0.008 ± 0.015		0.915
Tetradecenoylcarnitine	0.012 ± 0.003	0.011 ± 0.001		0.584
Hexadecanoylcarnitine	0.002 ± 0.004	0.041 ± 0.019	18.8	**0.007**
Hydroxyhexadecanoylcarnitine	0.000 ± 0.000	0.001 ± 0.002		0.356
Hexadecenoylcarnitine	0.000 ± 0.000	0.011 ± 0.014	*11.0*	0.196
Octadecanoylcarnitine	0.001 ± 0.003	0.015 ± 0.005	10.7	**0.004***
Octadecenoylcarnitine	0.000 ± 0.000	0.024 ± 0.029	*24.0*	0.153
Hydroxyoctadecenoylcarnitine	0.000 ± 0.000	0.001 ± 0.002		0.356
**Total Acylcarnitines**	**0.03 ± 0.02**	**0.13 ± 0.08**	**5.3**	**0.036**
3-Methylhistidine	0.000 ± 0.000	0.001 ± 0.001		0.136
Creatinine	0.000 ± 0.000	0.80 ± 1.60	*800*	0.356
Cystine	0.000 ± 0.000	0.014 ± 0.025	*14.0*	0.321
Kynurenine	0.037 ± 0.074	0.000 ± 0.000	*0.03*	0.356
trans-4-Hydroxyproline	0.063 ± 0.080	0.11 ± 0.05		0.327
**Total Amino Acid Related**	**0.10 ± 0.12**	**0.93 ± 1.6**	**9.3**	0.346
Asparagine	0.14 ± 0.16	0.066 ± 0.077		0.443
Cysteine	0.36 ± 0.06	0.43 ± 0.19		0.457
Leucine	0.21 ± 0.43	0.12 ± 0.24		0.721
**Total Amino Acids**	**0.71 ± 0.58**	**0.62 ± 0.34**	**0.9**	0.804
Chenodeoxycholic acid	0.000 ± 0.000	0.008 ± 0.005	8.0	**0.023**
Glycocholic acid	0.009 ± 0.006	0.43 ± 0.26	47.4	**0.018**
Glycochenodeoxycholic acid	0.005 ± 0.003	0.84 ± 0.38	167	**0.005***
Glycodeoxycholic acid	0.002 ± 0.004	0.021 ± 0.037	8.5	0.362
Glycolithocholic acid	0.000 ± 0.001	0.001 ± 0.001		0.299

Glycolithocholic acid sulfate	0.000 ± 0.000	0.002 ± 0.003		0.199
Taurocholic acid	0.000 ± 0.000	0.65 ± 0.64	*650*	0.089
Taurochenodeoxycholic acid	0.005 ± 0.004	1.4 ± 0.7	291	**0.005***
Taurodeoxycholic acid	0.001 ± 0.002	0.000 ± 0.000		0.356
**Total Bile Acids**	**0.023 ± 0.016**	**3.3 ± 1.4**	**146**	**0.003**
**Dopamine**	**0.009 ± 0.018**	**0.011 ± 0.022**		0.878
**Hexose**	**0.000 ± 0.000**	**307 ± 614**	**307**	0.356
**Cortisol**	**0.000 ± 0.000**	**0.002 ± 0.004**		0.356
**Dehydroepiandrosterone sulfate**	**0.005 ± 0.009**	**0.000 ± 0.000**	***0.2***	0.623
**Indoxylsulfate**	**0.002 ± 0.003**	**0.002 ± 0.003**		0.776
Ceramide (d16:1/18:0)	0.053 ± 0.005	0.083 ± 0.079		0.476
Ceramide (d16:1/20:0)	0.051 ± 0.008	0.076 ± 0.074		0.537
Ceramide (d16:1/22:0)	0.17 ± 0.02	0.13 ± 0.10		0.534
Ceramide (d16:1/23:0)	0.082 ± 0.004	0.041 ± 0.029	0.5	**0.029**
Ceramide (d16:1/24:0)	0.13 ± 0.02	0.046 ± 0.17	0.4	**0.001***
Ceramide (d18:1/14:0)	0.014 ± 0.029	0.062 ± 0.017	4.3	**0.029**
Ceramide (d18:1/16:0)	0.24 ± 0.03	0.53 ± 0.15	2.2	**0.009**
Ceramide (d18:1/18:0(OH))	0.038 ± 0.076	0.24 ± 0.10	6.4	**0.017**
Ceramide (d18:1/18:0)	0.10 ± 0.02	0.24 ± 0.24		0.310
Ceramide (d18:1/18:1)	0.006 ± 0.012	0.018 ± 0.014		0.250
Ceramide (d18:1/20:0(OH))	0.19 ± 0.01	0.55 ± 0.29	2.9	**0.045**
Ceramide (d18:1/20:0)	0.066 ± 0.006	0.14 ± 0.11		0.240
Ceramide (d18:1/22:0)	0.41 ± 0.08	0.44 ± 0.24		0.802
Ceramide (d18:1/23:0)	0.48 ± 0.12	0.35 ± 0.16		0.239
Ceramide (d18:1/24:0)	1.3 ± 0.3	0.73 ± 0.21	0.6	**0.019**
Ceramide (d18:1/24:1)	0.48 ± 0.04	1.3 ± 0.6	2.8	**0.022**
Ceramide (d18:1/25:0)	0.26 ± 0.02	0.25 ± 0.06		0.873
Ceramide (d18:1/26:0)	0.082 ± 0.056	0.034 ± 0.014		0.147
Ceramide (d18:1/26:1)	0.018 ± 0.008	0.030 ± 0.011		0.124
Ceramide (d18:2/16:0)	0.046 ± 0.007	0.089 ± 0.018	1.9	**0.004**
Ceramide (d18:2/18:0)	0.035 ± 0.003	0.057 ± 0.049		0.404
Ceramide (d18:2/18:1)	0.004 ± 0.005	0.007 ± 0.005		0.484
Ceramide (d18:2/20:0)	0.032 ± 0.007	0.056 ± 0.036		0.237

Ceramide (d18:2/22:0)	0.105 ± 0.022	0.095 ± 0.042		0.671
Ceramide (d18:2/23:0)	0.056 ± 0.016	0.031 ± 0.014		0.059
Ceramide (d18:2/24:0)	0.23 ± 0.06	0.11 ± 0.04	0.5	**0.012**
Ceramide (d18:2/24:1)	0.08 ± 0.01	0.17 ± 0.05	2.0	**0.011**
**Total Ceramides**	**4.8 ± 0.5**	**5.9 ± 2.4**	**1.2**	0.388
Cholesteryl ester 14:0	1.1 ± 2.1	0.000 ± 0.000		0.356
Cholesteryl ester 14:1	0.006 ± 0.012	0.000 ± 0.000		0.356
Cholesteryl ester 15:0	2.3 ± 0.5	1.3 ± 0.4	0.6	**0.014**
Cholesteryl ester 15:1	0.37 ± 0.10	0.17 ± 0.05	0.5	**0.011**
Cholesteryl ester 16:0	38.4 ± 3.7	22.3 ± 12.0	0.6	**0.043**
Cholesteryl ester 16:1	13.5 ± 8.3	14.5 ± 8.2		0.864
Cholesteryl ester 17:0	1.6 ± 0.5	0.65 ± 0.78		0.073
Cholesteryl ester 17:1	0.37 ± 0.27	0.37 ± 0.29		0.989
Cholesteryl ester 18:0	2.6 ± 0.6	1.3 ± 1.1		0.078
Cholesteryl ester 18:1	53.6 ± 8.7	29.1 ± 18.5		0.053
Cholesteryl ester 18:2	212 ± 17	80.3 ± 67.5	0.4	**0.009**
Cholesteryl ester 18:3	12.1 ± 1.7	4.4 ± 2.8	0.4	**0.004**
Cholesteryl ester 20:0	1.1 ± 0.6	0.56 ± 0.68		0.260
Cholesteryl ester 20:1	0.087 ± 0.175	0.000 ± 0.000	*0.01*	0.356
Cholesteryl ester 20:3	3.6 ± 0.6	0.86 ± 1.12	0.2	**0.005**
Cholesteryl ester 20:4	34.7 ± 7.8	14.1 ± 10.7	0.4	**0.021**
Cholesteryl ester 20:5	15.7 ± 5.7	2.4 ± 1.9	0.2	**0.004**
Cholesteryl ester 22:5	0.21 ± 0.25	0.000 ± 0.000	*0.005*	0.143
Cholesteryl ester 22:6	5.0 ± 1.3	1.4 ± 1.1	0.3	**0.005**
**Total Cholesteryl Esters**	**399 ± 18**	**174 ± 114**	**0.4**	**0.008**
Diacylglyceride (14:0_14:0)	0.54 ± 0.12	0.49 ± 0.19		0.698
Diacylglyceride (14:1_18:1)	0.000 ± 0.000	0.067 ± 0.133	*67*	0.356
Diacylglyceride (14:1_20:2)	0.010 ± 0.019	0.011 ± 0.013		0.931
Diacylglyceride (16:0_16:1)	0.085 ± 0.169	0.17 ± 0.34		0.674
Diacylglyceride (16:0_18:1)	1.0 ± 1.2	2.3 ± 0.4		0.076
Diacylglyceride (16:0_18:2)	1.1 ± 0.2	1.1 ± 0.5		0.978
Diacylglyceride (16:0_20:4)	0.000 ± 0.000	0.48 ± 0.96	*480*	0.356
Diacylglyceride (16:1_18:0)	0.000 ± 0.000	0.05 ± 0.10	*50*	0.356

Diacylglyceride (16:1_18:2)	0.37 ± 0.04	0.48 ± 0.09		0.055
Diacylglyceride (16:1_20:0)	0.048 ± 0.034	0.012 ± 0.013		0.097
Diacylglyceride (17:0_18:1)	0.087 ± 0.106	0.31 ± 0.02	3.6	**0.006**
Diacylglyceride (18:1_18:1)	0.60 ± 0.07	1.4 ± 0.3	2.3	**0.001***
Diacylglyceride (18:1_18:2)	1.6 ± 0.3	2.4 ± 1.0		0.188
Diacylglyceride (18:1_20:1)	0.000 ± 0.000	0.13 ± 0.16	*130*	0.134
Diacylglyceride (18:2_18:2)	1.2 ± 0.1	1.3 ± 0.8		0.745
Diacylglyceride (18:2_18:3)	0.28 ± 0.10	0.25 ± 0.06		0.653
Diacylglyceride (18:2_18:4)	0.077 ± 0.090	0.10 ± 0.11		0.754
Diacylglyceride (18:2_20:0)	0.000 ± 0.000	0.021 ± 0.041	*21*	0.356
Diacylglyceride (18:2_20:4)	0.000 ± 0.000	0.50 ± 0.58	*500*	0.134
Diacylglyceride (18:3_18:3)	0.10 ± 0.12	0.12 ± 0.04		0.741
Diacylglyceride (21:0_22:6)	0.74 ± 0.21	0.77 ± 0.34		0.906
Diacylglyceride O-(16:0_20:4)	0.005 ± 0.009	0.000 ± 0.000	*0.2*	0.356
**Total Diacylglycerides**	**7.8 ± 1.4**	**12.5 ± 1.1**	**1.6**	**0.002***
Dihexosylceramide (d18:1/14:0)	0.10 ± 0.02	0.15 ± 0.04	1.6	**0.042**
Dihexosylceramide (d18:1/16:0)	1.3 ± 0.3	2.7 ± 0.6	2.0	**0.007**
Dihexosylceramide (d18:1/18:0)	0.09 ± 0.01	0.13 ± 0.03	1.5	**0.039**
Dihexosylceramide (d18:1/20:0)	0.04 ± 0.00	0.06 ± 0.01	1.5	**0.030**
Dihexosylceramide (d18:1/22:0)	0.10 ± 0.01	0.18 ± 0.06	1.7	**0.042**
Dihexosylceramide (d18:1/24:0)	0.08 ± 0.01	0.17 ± 0.08		0.080
Dihexosylceramide (d18:1/24:1)	0.16 ± 0.02	0.64 ± 0.33	4.1	**0.028**
Dihexosylceramide (d18:1/26:0)	0.000 ± 0.000	0.004 ± 0.005		0.171
Dihexosylceramide (d18:1/26:1)	0.001 ± 0.002	0.008 ± 0.003	7.4	**0.009**
**Total Dihexosylceramides**	**1.9 ± 0.3**	**4.0 ± 1.1**	**2.1**	**0.008**
Dihydroceramide (d18:0/18:0(OH))	0.000 ± 0.000	0.05 ± 0.06	*50*	0.165
Dihydroceramide (d18:0/18:0)	0.003 ± 0.006	0.04 ± 0.05		0.232
Dihydroceramide (d18:0/20:0)	0.02 ± 0.03	0.05 ± 0.07		0.364
Dihydroceramide (d18:0/22:0)	0.09 ± 0.06	0.14 ± 0.11		0.376
Dihydroceramide (d18:0/24:0)	0.19 ± 0.05	0.14 ± 0.05		0.214
Dihydroceramide (d18:0/24:1)	0.12 ± 0.08	0.44 ± 0.34		0.111
Dihydroceramide (d18:0/26:1(OH))	0.04 ± 0.08	0.13 ± 0.26		0.520
**Total Dihydroceramides**	**0.45 ± 0.20**	**0.99 ± 0.61**	**2.2**	0.142

Arachidonic acid	0.000 ± 0.000	3.2 ± 1.9	3.2	**0.013**
Docosahexaenoic acid	0.25 ± 0.07	0.92 ± 0.32	3.7	**0.007**
Eicosapentaenoic acid	0.073 ± 0.085	0.15 ± 0.15		0.401
Octadecenoic acid	0.000 ± 0.000	14.6 ± 12.4	14.6	0.056
Octadecadienoate	0.19 ± 0.38	2.4 ± 0.98	12.6	**0.006***
Eicosenoic acid	0.015 ± 0.029	0.38 ± 0.62	25.9	0.284
Eicosadienoic acid	0.000 ± 0.000	0.14 ± 0.16	140	0.139
Eicosatrienoic acid	0.000 ± 0.000	0.077 ± 0.109	77.0	0.207
**Total Fatty Acids**	**0.5 ± 0.5**	**21.9 ± 15.6**	**41.6**	**0.034**
Hexosylceramide (d16:1/22:0)	0.018 ± 0.005	0.015 ± 0.006		0.533
Hexosylceramide (d16:1/24:0)	0.011 ± 0.003	0.014 ± 0.004		0.364
Hexosylceramide (d18:1/14:0)	0.003 ± 0.006	0.011 ± 0.009		0.233
Hexosylceramide (d18:1/16:0)	0.57 ± 0.11	0.88 ± 0.31		0.112
Hexosylceramide (d18:1/18:0)	0.090 ± 0.011	0.068 ± 0.012	0.8	**0.033**
Hexosylceramide (d18:1/18:1)	0.036 ± 0.007	0.053 ± 0.012		0.050
Hexosylceramide (d18:1/20:0)	0.14 ± 0.02	0.16 ± 0.04		0.480
Hexosylceramide (d18:1/22:0)	1.4 ± 0.2	1.4 ± 0.7		0.954
Hexosylceramide (d18:1/23:0)	0.69 ± 0.08	0.64 ± 0.29		0.766
Hexosylceramide (d18:1/24:0)	0.73 ± 0.10	0.85 ± 0.44		0.598
Hexosylceramide (d18:1/24:1)	2.0 ± 0.3	3.2 ± 1.6		0.195
Hexosylceramide (d18:1/26:0)	0.032 ± 0.008	0.042 ± 0.013		0.201
Hexosylceramide (d18:1/26:1)	0.023 ± 0.005	0.026 ± 0.008		0.630
Hexosylceramide (d18:2/16:0)	0.003 ± 0.004	0.003 ± 0.003		0.803
Hexosylceramide (d18:2/20:0)	0.001 ± 0.003	0.003 ± 0.003		0.591
Hexosylceramide (d18:2/22:0)	0.24 ± 0.03	0.20 ± 0.06		0.297
Hexosylceramide (d18:2/23:0)	0.11 ± 0.03	0.095 ± 0.015		0.553
Hexosylceramide (d18:2/24:0)	0.35 ± 0.06	0.37 ± 0.11		0.785
**Total Hexosylceramides**	**6.5 ± 0.9**	**8.0 ± 3.4**	**1.2**	0.415
Lysophosphatidylcholine a C16:0	14.4 ± 1.5	34.6 ± 12.5	2.4	**0.018**
Lysophosphatidylcholine a C16:1	0.14 ± 0.03	0.54 ± 0.28	3.8	**0.031**
Lysophosphatidylcholine a C17:0	0.30 ± 0.07	0.56 ± 0.33		0.177
Lysophosphatidylcholine a C18:0	9.8 ± 1.1	16.1 ± 4.2	1.7	**0.025**
Lysophosphatidylcholine a C18:1	1.8 ± 0.2	4.7 ± 1.5	2.5	**0.009**

Lysophosphatidylcholine a C18:2	1.0 ± 0.1	1.7 ± 0.9		0.189
Lysophosphatidylcholine a C20:3	0.06 ± 0.12	0.24 ± 0.17		0.123
Lysophosphatidylcholine a C20:4	0.30 ± 0.05	0.59 ± 0.28		0.096
Lysophosphatidylcholine a C24:0	0.048 ± 0.006	0.062 ± 0.016		0.164
Lysophosphatidylcholine a C26:0	0.045 ± 0.010	0.072 ± 0.032		0.167
Lysophosphatidylcholine a C26:1	0.008 ± 0.016	0.071 ± 0.021	9.2	**0.003***
Lysophosphatidylcholine a C28:0	0.000 ± 0.000	0.098 ± 0.074	10.0	**0.037**
Lysophosphatidylcholine a C28:1	0.14 ± 0.03	0.19 ± 0.10	1.4	0.371
**Total Lysophosphatidylcholines**	**28.1 ± 2.3**	**59.5 ± 19.7**	**2.1**	**0.019**
Phosphatidylcholine aa C24:0	0.004 ± 0.007	0.016± 0.015		0.179
Phosphatidylcholine aa C28:1	1.4 ± 0.2	1.9 ± 0.4		0.061
Phosphatidylcholine aa C30:0	1.6 ± 0.1	4.9 ± 1.5	3.1	**0.005**
Phosphatidylcholine aa C32:0	5.0 ± 0.5	28.6 ± 8.6	5.7	**0.002**
Phosphatidylcholine aa C32:1	3.8 ± 2.9	37.2 ± 25.9	9.7	**0.043**
Phosphatidylcholine aa C32:2	0.66 ± 0.11	2.7 ± 1.7	4.1	**0.049**
Phosphatidylcholine aa C32:3	0.14 ± 0.02	0.20 ± 0.10		0.264
Phosphatidylcholine aa C34:1	47.2 ± 9.0	192 ± 89.0	4.1	**0.018**
Phosphatidylcholine aa C34:2	79.0 ± 12.6	221 ± 135		0.081
Phosphatidylcholine aa C34:3	2.6 ± 0.5	8.6 ± 5.3		0.064
Phosphatidylcholine aa C34:4	0.30 ± 0.03	0.46 ± 0.31		0.332
Phosphatidylcholine aa C36:1	11.9 ± 1.3	29.0 ± 9.3	2.4	**0.011**
Phosphatidylcholine aa C36:2	51.6 ± 6.5	120.0 ± 37.0	2.3	**0.010**
Phosphatidylcholine aa C36:3	24.4 ± 2.7	52.3 ± 20.3	2.2	**0.034**
Phosphatidylcholine aa C36:4	32.0 ± 6.1	79.2 ± 50.5		0.113
Phosphatidylcholine aa C36:5	3.9 ± 1.1	4.2 ± 3.1		0.868
Phosphatidylcholine aa C36:6	0.13 ± 0.02	0.16 ± 0.11		0.716
Phosphatidylcholine aa C38:0	0.44 ± 0.01	0.96 ± 0.16	2.2	**0.001***
Phosphatidylcholine aa C38:3	11.1 ± 1.4	19.8 ± 6.0	1.8	**0.030**
Phosphatidylcholine aa C38:4	20.8 ± 5.3	52.6 ± 27.7		0.065
Phosphatidylcholine aa C38:5	8.9 ± 0.7	14.7 ± 7.3		0.155
Phosphatidylcholine aa C38:6	11.6 ± 2.0	21.8 ± 18.9		0.324
Phosphatidylcholine aa C40:2	0.064 ± 0.003	0.15 ± 0.03	2.4	**0.001***
Phosphatidylcholine aa C40:3	0.13 ± 0.01	0.27 ± 0.06	2.2	**0.004**

Phosphatidylcholine aa C40:4	0.69 ± 0.21	1.2 ± 0.3	1.7	**0.026**
Phosphatidylcholine aa C40:5	1.7 ± 0.4	3.0 ± 1.2		0.079
Phosphatidylcholine aa C40:6	4.3 ± 0.5	9.4 ± 7.1		0.201
Phosphatidylcholine aa C42:0	0.108 ± 0.004	0.23 ± 0.05	2.1	**0.002**
Phosphatidylcholine aa C42:1	0.053 ± 0.004	0.12 ± 0.02	2.3	**0.001***
Phosphatidylcholine aa C42:2	0.033 ± 0.038	0.12 ± 0.03	3.8	**0.010**
Phosphatidylcholine aa C42:4	0.045 ± 0.009	0.11 ± 0.02	2.4	**0.001***
Phosphatidylcholine aa C42:5	0.067 ± 0.013	0.19 ± 0.06	2.8	**0.009**
Phosphatidylcholine aa C42:6	0.063 ± 0.073	0.19 ± 0.05	3.0	**0.031**
Phosphatidylcholine ae C30:0	0.13 ± 0.02	0.36 ± 0.08	2.8	**0.001***
Phosphatidylcholine ae C30:1	0.000 ± 0.000	0.092 ± 0.085	*92*	0.073
Phosphatidylcholine ae C30:2	0.033 ± 0.006	0.045 ± 0.017		0.260
Phosphatidylcholine ae C32:1	0.81 ± 0.12	3.8 ± 1.3	4.7	**0.003**
Phosphatidylcholine ae C32:2	0.19 ± 0.03	0.92 ± 0.40	4.9	**0.011**
Phosphatidylcholine ae C34:0	0.42 ± 0.09	1.3 ± 0.3	3.2	**0.002**
Phosphatidylcholine ae C34:1	2.2 ± 0.3	9.9 ± 2.0	4.6	**0.0002***
Phosphatidylcholine ae C34:2	2.2 ± 0.5	5.2 ± 1.7	2.3	**0.016**
Phosphatidylcholine ae C34:3	1.5 ± 0.4	2.5 ± 0.6	1.7	**0.040**
Phosphatidylcholine ae C36:0	0.20 ± 0.02	0.67 ± 0.27	3.4	**0.013**
Phosphatidylcholine ae C36:1	2.3 ± 0.4	4.5 ± 2.7		0.147
Phosphatidylcholine ae C36:2	2.4 ± 0.5	5.8 ± 3.5		0.104
Phosphatidylcholine ae C36:3	1.3 ± 0.3	2.5 ± 0.6	1.9	**0.012**
Phosphatidylcholine ae C36:4	3.3 ± 1.1	6.4 ± 1.6	2.0	**0.018**
Phosphatidylcholine ae C36:5	2.2 ± 0.5	3.6 ± 0.5	1.7	**0.005**
Phosphatidylcholine ae C38:0	0.31 ± 0.02	0.40 ± 0.21		0.424
Phosphatidylcholine ae C38:1	0.077 ± 0.026	0.53 ± 0.13	6.9	**0.001***
Phosphatidylcholine ae C38:2	0.41 ± 0.03	0.95 ± 0.26	2.3	**0.007**
Phosphatidylcholine ae C38:3	1.1 ± 0.2	1.9 ± 0.8		0.120
Phosphatidylcholine ae C38:4	2.3 ± 0.9	4.4 ± 1.3	1.9	**0.037**
Phosphatidylcholine ae C38:5	2.9 ± 0.7	7.0 ± 1.9	2.4	**0.007**
Phosphatidylcholine ae C38:6	1.2 ± 0.1	2.0 ± 0.2	1.6	**0.0002***
Phosphatidylcholine ae C40:1	0.18 ± 0.02	0.23 ± 0.07	1.3	0.253
Phosphatidylcholine ae C40:2	0.54 ± 0.03	0.98 ± 0.41	1.8	0.078

Phosphatidylcholine ae C40:3	0.45 ± 0.07	0.91 ± 0.35	2.0	**0.043**
Phosphatidylcholine ae C40:4	0.55 ± 0.15	0.97 ± 0.21	1.8	**0.016**
Phosphatidylcholine ae C40:5	0.76 ± 0.12	1.4 ± 0.2	1.8	**0.003**
Phosphatidylcholine ae C40:6	0.65 ± 0.08	1.2 ± 0.4	1.9	**0.029**
Phosphatidylcholine ae C42:1	0.091 ± 0.007	0.15 ± 0.04	1.6	**0.041**
Phosphatidylcholine ae C42:2	0.100 ± 0.006	0.16 ± 0.02	1.6	**0.003**
Phosphatidylcholine ae C42:3	0.15 ± 0.01	0.26 ± 0.05	1.8	**0.006**
Phosphatidylcholine ae C42:4	0.18 ± 0.04	0.39 ± 0.07	2.2	**0.002**
Phosphatidylcholine ae C42:5	0.12 ± 0.24	1.1 ± 0.3	9.3	**0.001***
Phosphatidylcholine ae C44:3	0.036 ± 0.025	0.069 ± 0.016		0.066
Phosphatidylcholine ae C44:4	0.070 ± 0.049	0.15 ± 0.03	2.1	**0.033**
Phosphatidylcholine ae C44:5	0.28 ± 0.08	0.82 ± 0.19	3.0	**0.002**
Phosphatidylcholine ae C44:6	0.22 ± 0.04	0.56 ± 0.12	2.5	**0.002**
**Total Phosphatidylcholines**	**357 ± 31.0**	**981 ± 411**	**2.7**	**0.023**
Hydroxysphingomyelin C14:1	2.2 ± 0.6	2.6 ± 1.3		0.604
Hydroxysphingomyelin C16:1	1.2 ± 0.3	1.1 ± 0.8		0.899
Hydroxysphingomyelin C22:1	4.2 ± 1.0	2.9 ± 1.4		0.185
Hydroxysphingomyelin C22:2	2.7 ± 0.5	2.7 ± 1.3		0.923
Hydroxysphingomyelin C24:1	0.35 ± 0.10	0.47 ± 0.15		0.233
Sphingomyelin C16:0	44.8 ± 8.9	61.0 ± 14.4		0.105
Sphingomyelin C16:1	5.2 ± 0.9	7.9 ± 1.0	1.5	**0.008**
Sphingomyelin C18:0	9.0 ± 1.6	8.9 ± 5.3		0.968
Sphingomyelin C18:1	3.6 ± 0.6	3.4 ± 1.9		0.917
Sphingomyelin C20:2	0.12 ± 0.02	0.23 ± 0.14		0.180
Sphingomyelin C24:0	6.7 ± 1.4	6.4 ± 2.2		0.832
Sphingomyelin C24:1	13.5 ± 1.2	24.1 ± 6.6	1.8	**0.020**
Sphingomyelin C26:0	0.043 ± 0.005	0.061 ± 0.022		0.170
Sphingomyelin C26:1	0.083 ± 0.006	0.17 ± 0.07	2.1	**0.046**
**Total Sphingomyelins**	**93.6 ± 16.1**	**122 ± 32.0**	**1.3**	0.168
Triacylglycerides C44:1	0.20 ± 0.22	0.61 ± 0.75	3.0	0.335
Triacylglycerides C44:2	0.09 ± 0.10	0.31 ± 0.41	3.5	0.332
Triacylglycerides C46:2	0.34 ± 0.21	1.84 ± 2.40	5.4	0.260
Triacylglycerides C48:0	2.00 ± 1.50	7.01 ± 7.65	3.5	0.246

Triacylglycerides C48:1	3.86 ± 2.13	16.72 ± 18.78	4.3	0.222
Triacylglycerides C48:2	2.52 ± 0.97	14.43 ± 16.72	*5.7*	0.205
Triacylglycerides C48:3	0.54 ± 0.13	2.81 ± 3.19	*5.2*	0.205
Triacylglycerides C49:1	0.44 ± 0.09	1.18 ± 0.68	2.7	0.075
Triacylglycerides C49:2	0.24 ± 0.08	0.93 ± 0.71	3.8	0.103
Triacylglycerides C50:0	2.60 ± 1.48	8.33 ± 7.25	3.2	0.173
Triacylglycerides C50:1	9.77 ± 2.52	39.62 ± 30.62	4.1	0.100
Triacylglycerides C50:2	9.15 ± 1.41	46.31 ± 37.70	*5.1*	0.096
Triacylglycerides C50:3	4.73 ± 0.55	21.31 ± 17.13	4.5	0.101
Triacylglycerides C50:4	0.92 ± 0.20	3.17 ± 2.45	3.4	0.117
Triacylglycerides C51:1	0.71 ± 0.18	1.84 ± 0.70	2.6	**0.020**
Triacylglycerides C51:2	0.92 ± 0.17	2.70 ± 0.97	2.9	**0.011**
Triacylglycerides C51:3	0.53 ± 0.13	1.43 ± 0.63	2.7	**0.033**
Triacylglycerides C51:4	0.18 ± 0.04	0.45 ± 0.23	2.5	0.059
Triacylglycerides C51:5	0.05 ± 0.00	0.14 ± 0.07	2.8	**0.038**
Triacylglycerides C52:1	0.02 ± 0.03	0.19 ± 0.15	*12.6*	0.060
Triacylglycerides C52:2	19.60 ± 2.02	66.60 ± 26.08	3.4	**0.012**
Triacylglycerides C52:3	14.70 ± 2.92	42.43 ± 14.87	2.9	**0.011**
Triacylglycerides C52:4	6.17 ± 1.60	14.79 ± 7.17	2.4	0.057
Triacylglycerides C52:5	1.55 ± 0.37	3.51 ± 1.94	2.3	0.094
Triacylglycerides C52:6	0.32 ± 0.08	0.67 ± 0.41	2.1	0.149
Triacylglycerides C53:3	0.79 ± 0.21	1.86 ± 0.62	2.4	**0.017**
Triacylglycerides C53:4	0.36 ± 0.09	0.85 ± 0.41	2.4	0.057
Triacylglycerides C53:5	0.12 ± 0.03	0.27 ± 0.13	2.3	0.066
Triacylglycerides C53:6	0.06 ± 0.01	0.16 ± 0.04	2.5	**0.005**
Triacylglycerides C54:1	0.39 ± 0.12	1.33 ± 0.79	3.4	0.059
Triacylglycerides C54:2	2.55 ± 0.33	10.08 ± 4.89	4.0	**0.022**
Triacylglycerides C54:3	4.62 ± 0.56	18.77 ± 6.23	4.1	**0.004**
Triacylglycerides C54:4	3.13 ± 0.44	9.20 ± 3.09	2.9	**0.008**
Triacylglycerides C54:5	2.14 ± 0.31	4.95 ± 2.38	2.3	0.058
Triacylglycerides C54:6	0.92 ± 0.18	1.87 ± 0.98	2.0	0.103
Triacylglycerides C54:7	0.49 ± 0.19	0.91 ± 0.51	1.8	0.174
Triacylglycerides C55:6	0.00 ± 0.00	0.00 ± 0.00	2.3	**0.016**

Triacylglycerides C55:7	0.02 ± 0.00	0.05 ± 0.01	2.6	**0.001***
Triacylglycerides C55:8	0.02 ± 0.01	0.05 ± 0.02	2.4	**0.015**
Triacylglycerides C55:9	0.01 ± 0.00	0.02 ± 0.01	1.9	0.072
Triacylglycerides C56:6	0.96 ± 0.24	1.92 ± 0.69	2.0	**0.040**
Triacylglycerides C56:7	0.85 ± 0.27	1.35 ± 0.49	1.6	0.123
Triacylglycerides C56:8	0.52 ± 0.23	0.67 ± 0.27	1.3	0.414
Triacylglycerides C56:9	0.21 ± 0.11	0.28 ± 0.15	1.3	0.518
**Total Triglycerides**	**100 ± 3**	**354 ± 202**	**3.5**	**0.046**
Trihexosylceramide (d18:1/16:0)	0.67 ± 0.10	1.4 ± 0.9		0.161
Trihexosylceramide (d18:1/18:0)	0.069 ± 0.018	0.087 ± 0.059		0.578
Trihexosylceramide (d18:1/24:1)	0.22 ± 0.04	0.96 ± 0.96		0.174
Trihexosylceramide (d18:1/26:1)	0.015 ± 0.006	0.036 ± 0.033		0.255
Trihexosylceramide (d18:1/20:0)	0.004 ± 0.001	0.006 ± 0.005		0.380
Trihexosylceramide (d18:1/22:0)	0.049 ± 0.011	0.10 ± 0.08		0.237
**Total Trihexosylceramides**	**1.0 ± 0.1**	**2.6 ± 2.0**	**2.5**	0.176

Data expressed as mean (μM) ± standard deviation. Values were normalized to apolipoprotein B content. P values <0.05 are in bold. Ratios of LP-Z/LDL in italics were estimated for lipids or metabolites that showed ≥5-fold increases or decreases despite a p-value that did not reach significance due to high variability in the small number of samples. The p-value threshold with Bonferroni correction is 0.003 for the 16 lipid/metabolite classes in Figure 5, and for each lipid species are as follows: Acylcarnitines = 0.004; Amino Acid Related = 0.010; Amino Acids = 0.017; Bile Acids = 0.005; Biogenic Amines = 0.05; Carbohydrates and Related = 0.05; Ceramides = 0.002; Cholesteryl Esters = 0.003; Diacylglycerides = 0.002; Dihexosylceramides = 0.006; Dihydroceramides = 0.007; Fatty Acids = 0.006; Hexosylceramides = 0.003; Lysophosphatidylcholines = 0.004; Phosphatidylcholines = 0.001; Sphingomyelins = 0.004; Triacylglycerides = 0.0011; Trihexosylceramides = 0.008. p-values less than these thresholds are considered statistically significant. *Significant when taking into account Bonferroni correction.
